# Space, time and number: common coding mechanisms and interactions between domains

**DOI:** 10.1007/s00426-021-01503-8

**Published:** 2021-03-23

**Authors:** Deborah J. Serrien, Michiel M. Spapé

**Affiliations:** 1 School of Psychology, University of Nottingham, Nottingham, UK; 2 Department of Psychology and Logopedics, University of Helsinki, Helsinki, Finland

## Abstract

Space, time and number are key dimensions that underlie how we perceive, identify and act within the environment. They are interconnected in our behaviour and brain. In this study, we examined interdependencies between these dimensions. To this end, left- and right-handed participants performed an object collision task that required space–time processing and arithmetic tests that involved number processing. Handedness of the participants influenced collision detection with left-handers being more accurate than right-handers, which is in line with the premise that hand preference guides individual differences as a result of sensorimotor experiences and distinct interhemispheric integration patterns. The data further showed that successful collision detection was a predictor for arithmetic achievement, at least in right-handers. These findings suggest that handedness plays a mediating role in binding information processing across domains, likely due to selective connectivity properties within the sensorimotor system that is guided by hemispheric lateralisation patterns.

## Introduction

In everyday life, we often interact with moving objects, such as catching a ball or crossing a road. Crucial to these sensorimotor activities is the ability to predict the trajectory of the moving objects and the changes of their position over time (Enns & Lleras, 2008; Senot, et al. 2003). To implement these predictions, the brain uses a range of quantitative inputs, such as spatial, temporal and numerical information. Moreover, space–time–number represent essential dimensions that can be encoded through all sensory modalities (Burr, et al. 2010). These dimensions further demonstrate associations, such as the SNARC effect, that captures number–space interactions with faster responses occurring to smaller/larger numbers on the left/right side of space due to a representation of increasing numerical value from left to right (Dehaene, et al. 1993).

To account for these interdependencies, Walsh (2003) argued for a magnitude system that involves processing of dimensional magnitudes and their interactions (Bonato, et al. 2012; Burr, et al. 2010; Dehaene & Brannon, 2001; Fabbri, et al. 2013; Hayashi, et al. 2013). Furthermore, the proposed ATOM model (A Theory of Magnitude) underlines that actions are instrumental in establishing the magnitude system, with parietal circuitry providing a neural platform to exchange information (Walsh, 2003). That is, it is through actions that associations between magnitudes are learned for example that larger objects tend to be heavier than smaller ones. Thus, the magnitude system ties interactions between dimensions such that ‘more’ in a dimension couples with ‘more’ in another dimension. The origin of these interactions is that they reflect innate mappings or developmental processes, although both types of mechanisms could influence one another with innate pathways being influenced by early experiences and learned processes by innate constraints (De Hevia, et al. 2014; Stanescu-Cosson, et al. 2000; Walsh, 2003). Besides innate and developmental systems, attentional processes also play an important role. For example, attention can be directed towards specific task features, such as a location in space or a moment in time, which accordingly supports behavioural performance (Coull & Nobre, 1998; Dehaene, et al. 2003).

According to current viewpoints, a dimension could emerge from another, resulting in functional similarities and dependencies in computational and neural mechanisms. One particular hypothesis is that space serves as a foundation for the dimensions that are conceptually more abstract, such as time and number (Bonn & Cantlon, 2012). Thus, magnitude representations with a common code could evolve due to the substrates that already exist for the space dimension. Of note, however, is that the existence of dimensional interactions does not imply that these are equal in strength. For example, there is evidence that space–time interactions are strongest due to the specialisation of the magnitude system for sensorimotor actions, followed by space–number and time–number interactions (Bueti & Walsh, 2009).

Taking into account that the magnitude system is established through actions, the question emerges about the effect of sensorimotor experiences. That is, it can be argued that magnitude representations would differ if individuals interact in different ways with the environment due to inherent biases. Handedness is such a characteristic that captures asymmetry of movement control and expresses how individuals use their hands during manual activities. Research has shown that handedness not only affects the sensorimotor control mechanisms (Klöppel, et al. 2007; Pool, et al. 2014; Reid & Serrien, 2014; Serrien, et al. 2012) but also influences how individuals attend to and respond to the environment. In other words, handedness influences a range of abilities that involves visuospatial functions (Bareham, et al. 2015; Hécaen & Sauguet, 1971; O’Regan & Serrien, 2018; Vogel, et al. 2003), attentional regulation in space and time (Buckingham et al., 2009; O’Regan, et al. 2017) and visual processing in perihand space (Le Bigot & Grosjean, 2012). Combined, these findings illustrate that handedness has a widespread impact on the processing requirements of space and time for meeting behavioural goals.

The aim of the present experiment is to examine how space–time processing naturally connects with number processing, based on the proposed interdependencies between the dimensions of space–time–number. In this respect, a valuable experimental approach is to study a functional effect at the level of the behavioural outputs. *First*, we use an object collision task that requires participants to predict whether moving objects will collide with one another or not at a specific moment in time. To be successful, an accurate estimation of the moving objects over time is required (O’Reilly, et al. 2008; Proffitt & Gilden, 1989). It involves information about the path of the objects in space which is strongly linked with spatial coordinates, whereas the velocity with which the objects move implies position changes in temporal coordinates. From a neural viewpoint, previous work has shown that collision detection associates with the left inferior parietal cortex (Assmuss, et al. 2003). *Second*, we include arithmetic tests that require the use of numerical information processing established by operator-dependent rules (Friedrich & Friederici, 2009). We use tests with different types of arithmetic operations; additions, subtractions, and multiplications. Neurally, research has shown that tasks that involve numbers and calculations involve bilateral inferior parietal activity as a function of the arithmetic operation (Arsalidou & Taylor, 2011), albeit with a key involvement of the left hemisphere (Dehaene, et al. 1993). *Third*, we study left- and right-handers based on the premise that handedness introduces distinct sensorimotor experiences that affect the processing demands. We also conduct an evaluation of the participants’ personality traits as a relationship between handedness and negative affect has been proposed due to an underlying influence of the right hemisphere (Sutton & Davidson, 1997) with left- as compared to right-handers obtaining higher self-reported levels of behavioural inhibition (Hardie & Wright, 2014).

In the present work, we argue that the space–time calculations of the object collision task associate with arithmetic computations due to shared neural mechanisms. We further hypothesise that the participants’ handedness guides the processing demands as a result of their sensorimotor experiences and interactions with objects. Combined, insights into individual differences of handedness and interdependencies across space–time–number processing will be valuable to increase our understanding of common mechanisms that underlie our behaviour.

## Methods

### Participants

There were 37 participants in this study (M_age_ = 20.7 years, SE_age_ = 0.6), including 19 left-handers and 18 right-handers. They reported no history of neurological or psychiatric conditions as evaluated by a standardised questionnaire, and had normal or corrected-to-normal vision. Participants gave written consent prior to the start of the experiment in accordance with the Declaration of Helsinki. The study was approved by the School of Psychology Ethics Committee.

### Handedness questionnaire

To characterise handedness, participants completed a 15-item handedness questionnaire that measured hand preference for manipulation tasks (i.e., write a letter, use spoon, use toothbrush, throw ball to hit target, use a comb, hold racquet, hold needle when sewing, draw a picture, use computer mouse, open lid from can, hold knife to cut, peel an apple, use scissors, deal cards, use eraser).

The handedness questionnaire used a 5-point Likert scale that varied between always left and always right. The score per item was calculated with a value of 0 (always left), 1 (usually left), 2 (both equally), 3 (usually right) or 4 (always right). For each participant, the scores of the items were summed, divided by the maximum score of the questionnaire, and multiplied by 100. This provided a laterality index of handedness that varied between 0 (extreme left-handedness) and 100 (extreme right-handedness). The writing hand was also included as a condition for handedness as most people will categorise their handedness on the basis of their writing hand (Perelle & Ehrman, 2005).

### Reinforcement sensitivity theory personality questionnaire (RST-PQ)

To capture personality traits, participants completed subtests of the RST-PQ (Corr & Cooper, 2016), which rely on the premise that individual differences emerge from neurobiological systems that are specialised in detecting, processing, and responding to stimuli. In particular, the Behavioural Inhibition System (BIS) engages risk assessment and inhibits behaviour in response to goal conflict, resulting in anxiety and behavioural avoidance. In contrast, the Behavioural Approach System (BAS) facilitates goal-directed activity and positive emotions, leading to optimistic mood and achieved goals. The questionnaire covered BIS activity with 23 items and BAS activity with 32 items; i.e., biased attention towards reward interest (BAS-RI with 7 items), goal drive persistence (BAS-GDR with 7 items), impulsivity (BAS-IMP with 8 items) and reward reactivity (BAS-RR with 10 items).

The questionnaire used a 4-point Likert scale for accuracy of statements, ranging between 1 (not at all) and 4 (highly accurate). For each sub-test, the ratings were summed across items to provide a total score. High scores indicated increased sensitivity of a given neurobiological system.

### Object collision task

**Fig. 1:**
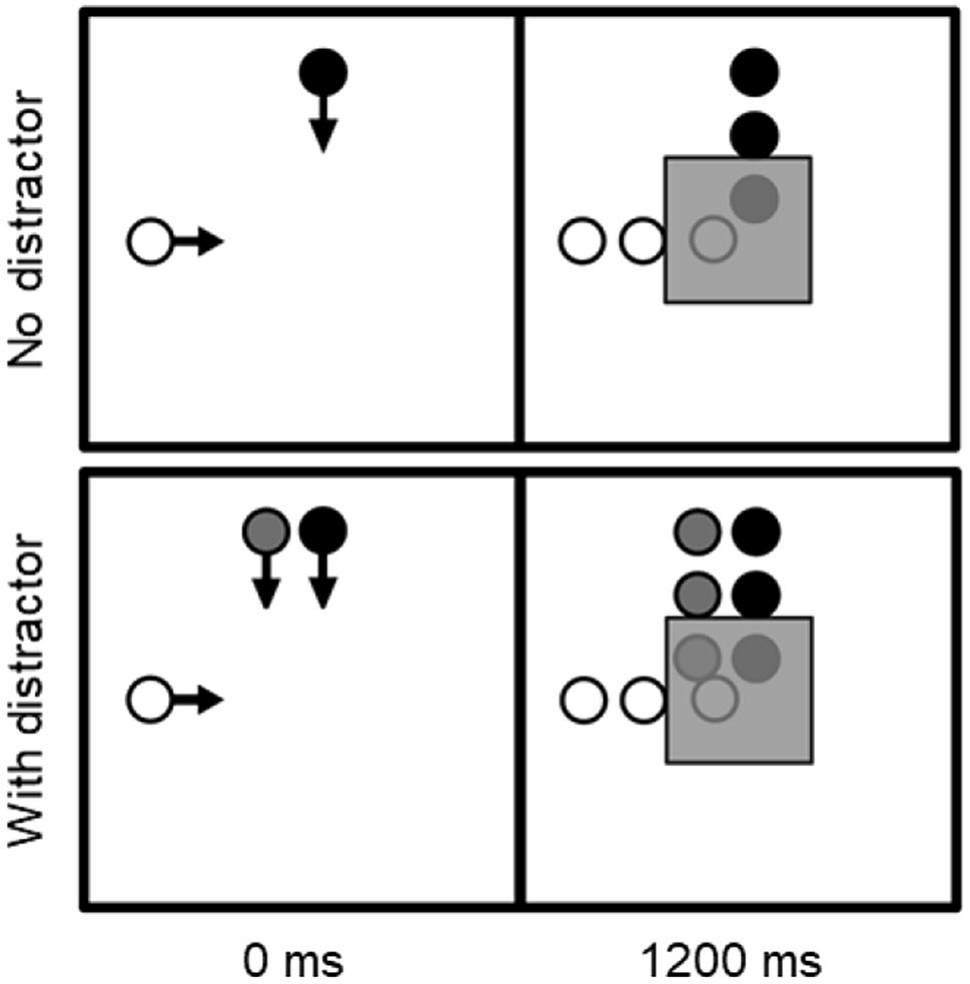
Collision task without and with distractor. Left side: after disappearance of the fixation cross, the task starts with the black and white objects moving towards the centre. Right side: after 1200 ms, these objects disappear behind a mask while their final trajectories are hidden from view. In the collision task with distractor, the grey object (distractor) moves along the black object on the side nearest to the white object

Participants were seated at a viewing distance of 70 cm from a computer monitor. The trial presentation is illustrated in Fig. 1. Each trial started with the presentation of a fixation cross that lasted 1000 ms followed by the appearance of a black and white object, either 3.6º on the upper, lower, left or right side of the screen’s centre. Thereafter, the perpendicular presented objects with a diameter of 0.4° would start to move in straight lines with a constant speed of 2.8 or 5.6°/s towards the screen’s centre, resulting in collision and non-collision events. As soon as the objects started to move, the participants were required to decide whether the objects would collide (target hit) or not collide (target miss) behind a mask that had a height and width of 3.6°. This point of collision which occurred 1300 ms after onset is not shown to the participants as the mask would hide the final trajectories of the objects from 1200 ms after onset. After another 700 ms or until the participants made a response, a blank screen occurred that marked the end of the trial. In 33% of the trials, a third grey object (distractor) would move with a similar speed alongside the black object towards the mask. These trials were included to influence attentional selection to the relevant objects. The performance conditions (without distractor vs. with distractor) and type of collision (target hit vs. target miss) were randomised. There were 32 trials per performance condition, resulting in a total of 128 trials. Participants were asked to respond as fast and as accurate as possible in their decision-making using keys allocated to the index and middle fingers of the left or right hand (counterbalanced). Before the start of the experiment, a training session with feedback was provided, and there were short breaks throughout the experiment. The trial sequence and data collection were implemented using e-Prime.

The measurements of the task were collision detection time (ms) and accuracy (%). The collision detection time comprised the time period between initiation of the moving objects and key press responses whereas the collision detection accuracy referred to correctly confirmed collisions on contact trials and correctly rejected collisions on no contact trials, and represented a key measurement that captures the ability to predict the collision event at a precise moment in time. We also calculated the balanced integration score to obtain a composite evaluation of both measurements. This index integrates reaction time and accuracy with equal weighting and is considered beneficial as compared to other methods that assess speed-accuracy trade-offs (Liesefeld & Janczyk, 2019; Vandierendonck, 2017). The balanced integration score is calculated by independently standardising the reaction times and percentage correct responses to bring them onto the same scale, and then subtracting one standardised score from the other. Its interpretation is in terms of performance above or below average, and therefore measures relative performance—for example, whether one group of participants is more successful than another group, or, whether one condition is more difficult than another condition.

### Arithmetic tests

Participants were asked to answer a series of arithmetic operations, i.e., additions, subtractions, multiplications using pen and paper. There were two lists consisting of 10 problems for each arithmetic operation, which were presented separately. For additions and subtractions, the problems involved double- and single-digit operands (64–7) or two double-digit operands (69 + 15) whereas for multiplications, the problems involved single-digit operands (7 × 4) or double- and single-digit operands (92 × 3), excluding 0 and 1 as one of the operands. Across the arithmetic tests, the problems required a combination of memory retrieval and computation. Participants were asked to answer the problems as fast and as accurate as possible. As a control condition, a number copying tasks were conducted. This consisted of copying a list of 20 numbers (four lists of five numbers) that involved double- or triple-digit operands. Short breaks between the tests were provided.

The measurements of the task were arithmetic performance time (s) and accuracy (%) for each arithmetic operation in addition to the number copying time (s) per list of five numbers.

### Analysis

The object collision measurements were analysed by means of 2 × 2 mixed-design ANOVAs (Handedness Group; left- vs. right-handers and Distractor Presence; with vs. without distractor). Secondary analyses assessing the start position of the objects or their speed did not show any significant effects, *p* > 0.05. The arithmetic measurements were analysed by means of 2 × 3 mixed-design ANOVAs (Handedness Group; left- vs. right-handers and Arithmetic Operation; additions vs. subtractions vs. multiplications). Frequency analyses were conducted by means of chi-square tests. The number copying measurement and the personality questionnaire scores were analysed by means of independent *t* tests on Handedness Group. A simple linear regression analysis was conducted to assess whether space–time detection of object collision predicted mathematical achievement. Initial checks showed that the Durbin–Watson test indicated no concern for autocorrelation, with data homoscedasticity and a normal probability plot of the residuals**.** Mean ± SE is reported. Bonferroni correction was made for multiple comparisons, where appropriate.

## Results

### Handedness questionnaire

The laterality index obtained from the handedness questionnaire was used to classify the participants, resulting into 19 left-handers (LI = 19 ± 3%, age = 23 ± 1y, 18 females) and 18 right-handers (LI = 93 ± 2%, age = 19 ± 1y, 11 females).

### Personality questionnaire

**Fig. 2:**
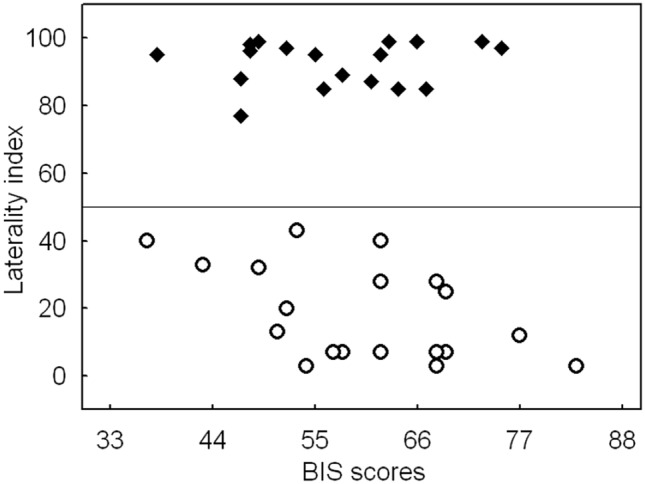
Scatter plot of the BIS scores obtained from the personality questionnaire (RST-PQ) as a function of the participants’ laterality index from the handedness questionnaire. The laterality index varied between 0 (extreme left-handedness) and 100 (extreme right-handedness). The middle line exemplifies a score of 50 (ambidextrous)

The total RST-PQ scores showed no significant difference between left-handers (*M* = 141.5 ± 3.7) and right-handers (*M* = 138.1 ± 3.2), *p* > 0.05. Additional analyses for the subtests revealed no significant differences between left- and right-handers for BIS (*M* = 60.1 ± 2.6 and 57.2 ± 2.4), BAS-RI (*M* = 17.4 ± 1.0 and 17.4 ± 0.8), BAS-GDR (*M* = 19.5 ± 0.6 and 17.9 ± 0.7), BAS-IMP (*M* = 16.9 ± 1.0 and 18.6 ± 1.3), BAS-RR (*M* = 27.5 ± 1.3 and 26.9 ± 1.0), all *p* > 0.05. In previous work, differences in negative affect have been associated with handedness (Hardie & Wright, 2014). However, we observed no indication of a significant shift in our sample, as shown in Fig. 2 which illustrates the participants’ BIS scores alongside their laterality index.

### Object collision task

For collision detection time, the ANOVA analysis demonstrated a significant main effect of Distractor Presence, *F*(1, 35) = 28.1, *p* < 0.01, ηp^2^ = 0.44, indicating that decision time was longer in the presence of distractors (*M* = 1159 ± 42 ms) than without distractors (*M* = 1096 ± 36 ms). No other effects were significant, *p* > 0.05.

**Fig. 3:**
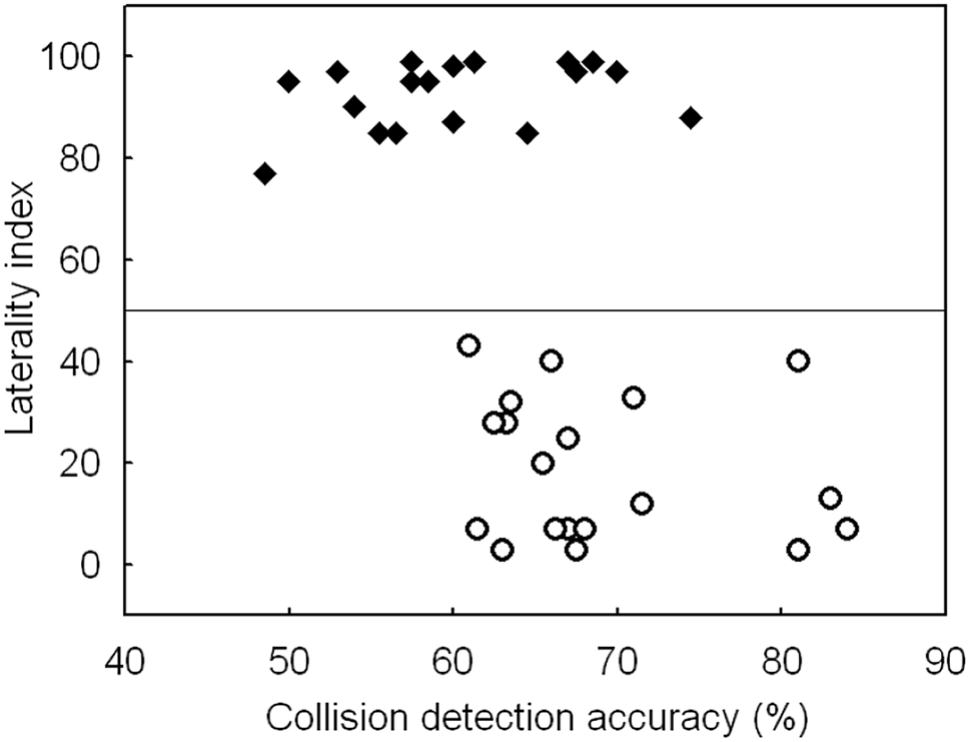
Scatter plot of the collision detection accuracy scores as a function of the participants’ laterality index from the handedness questionnaire. The accuracy scores represent the combined collision conditions. The laterality index varied between 0 (extreme left-handedness) and 100 (extreme right-handedness). The middle line exemplifies a score of 50 (ambidextrous)

For collision detection accuracy, the ANOVA analysis pointed to a significant main effect of Handedness Group, *F*(1, 35) = 13.5, *p* < 0.01, ηp^2^ = 0.28, showing that left-handers (*M* = 69 ± 2%) detected object collisions more accurately than right-handers (*M* = 60 ± 2%). No other effects were significant, *p* > 0.05. Figure 3 provides details about the collision detection accuracy of the participants alongside their laterality index, and shows that left-handers were more accurate performers than right-handers.

**Fig. 4:**
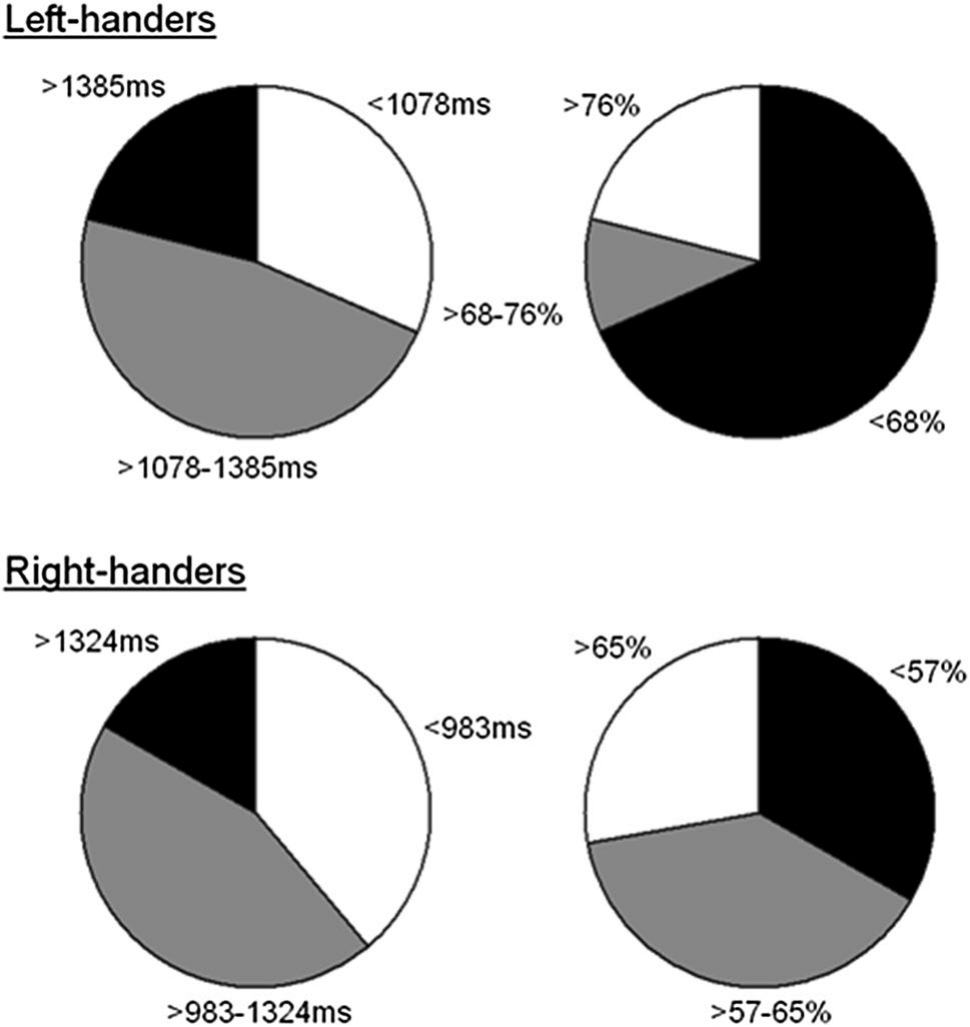
Categorisation of the collision detection times (left side) and accuracy scores (right side) for the left- and right-handers. The colour coding across the measurements indicates the performance level: superior (white), intermediate (grey) and inferior (black)

To portray more in detail the collision task for both handedness groups, Fig. 4 presents a categorisation of frequency counts of the participants according to three performance intervals: superior (i.e., fast/accurate performers), intermediate (in-between performers), and inferior (slow/inaccurate performers). For the collision detection time (left-sided panels), the data revealed that there were no significant group differences for the performance intervals, *p* > 0.05. Of note is that distractor presence did not impact the performance intervals (*p* > 0.05).

For collision detection accuracy (right-sided panels), the data indicated that both groups performed distinctively for the inferior and intermediate intervals, *χ*^2^1,_*N*=37_ = 11.45, *p* < 0.001; and *χ*^2^1, _*N*=*37*_ = 14.58, *p* < 0.0001. There was no difference for the superior interval, *p* > 0.05. Performing with or without distractor did not affect any of the intervals (*p* > 0.05).

The balanced integration score revealed a significant main effect of Handedness Group, *F*(1, 35) = 4.2, *p* < 0.05, η*p*^2^ = 0.11 with left-handers (0.28 ± 0.16) being more successful than right-handers (− 0.33 ± 0.28). There was also a significant main effect of Distractor Presence. *F*(1, 35) = 5.8, *p* < 0.05, η*p*^2^ = 0.14, indicating a stronger performance without distractor (0.12 ± 0.17) than with distractor (− 0.16 ± 0.16).

### Arithmetic tests

For arithmetic performance time, the ANOVA analysis revealed a significant main effect of Arithmetic Operation, *F*(2, 70) = 19.9 *p* < 0.01, η*p*^2^ = 0.36**,** demonstrating that additions (*M* = 80 ± 5 s) were performed fastest followed by subtractions (*M* = 117 ± 9 s) and multiplications (*M* = 151 ± 15 s). Post hoc comparisons demonstrated that all tests differed from one another, *p* < 0.01.

For arithmetic performance accuracy, the ANOVA analysis showed a significant main effect of Arithmetic Operation, *F*(2, 70) = 19.3, *p* < 0.01, η*p*^2^ = 0.35, revealing that additions (*M* = 95 ± 1%) obtained highest accuracy followed by subtractions (M = 90 ± 1%) and multiplications (*M* = 85 ± 2%). Post hoc comparisons demonstrated that all tests differed from one another, *p* < 0.01.

**Fig. 5:**
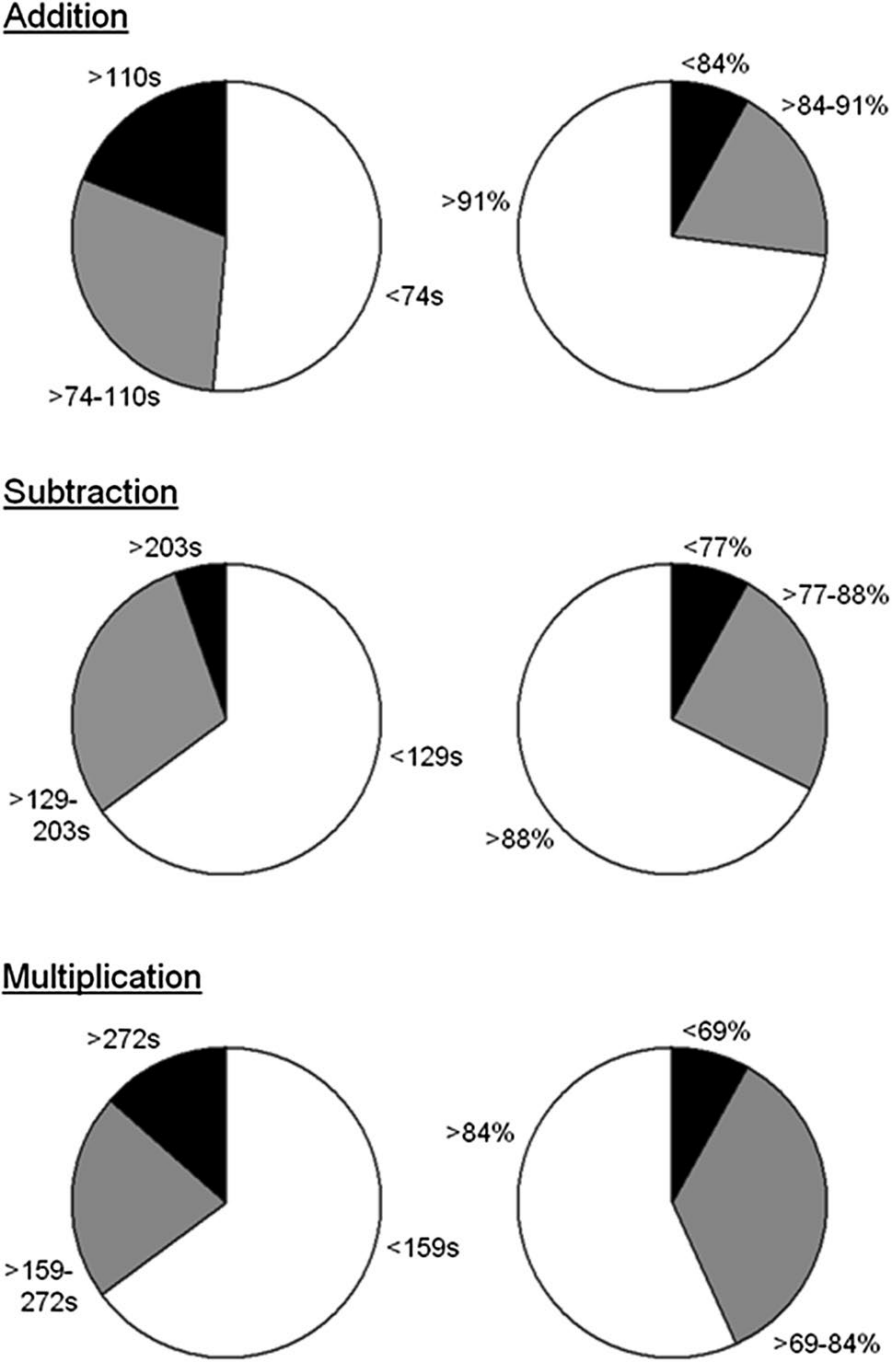
Categorisation of the arithmetic performance times (left side) and accuracy scores (right side) for additions, subtractions and multiplications. The colour coding across the measurements indicates the performance level: superior (white), intermediate (grey) and inferior (black)

To illustrate the arithmetic tests in more detail, Fig. 5 presents a frequency count for the arithmetic operations according to three performance intervals: superior (i.e., fast/accurate performers), intermediate (in-between performers), and inferior (slow/inaccurate performers). Across the measurements of performance time (left-sided panels) and accuracy (right-sided panels), the data illustrate that ± 30% of the participants performed in the intermediate range whereas ± 60% corresponded to fast/accurate performers and ± 10% were slow/inaccurate performers.

For the number copying task, the analysis demonstrated no significant difference between left-handers (*M* = 7.7 ± 0.3 s) and right-handers (*M* = 7.5 ± 0.3 s), *p* > 0.05.

### Object collision task and arithmetic tests: regression analysis

**Fig. 6:**
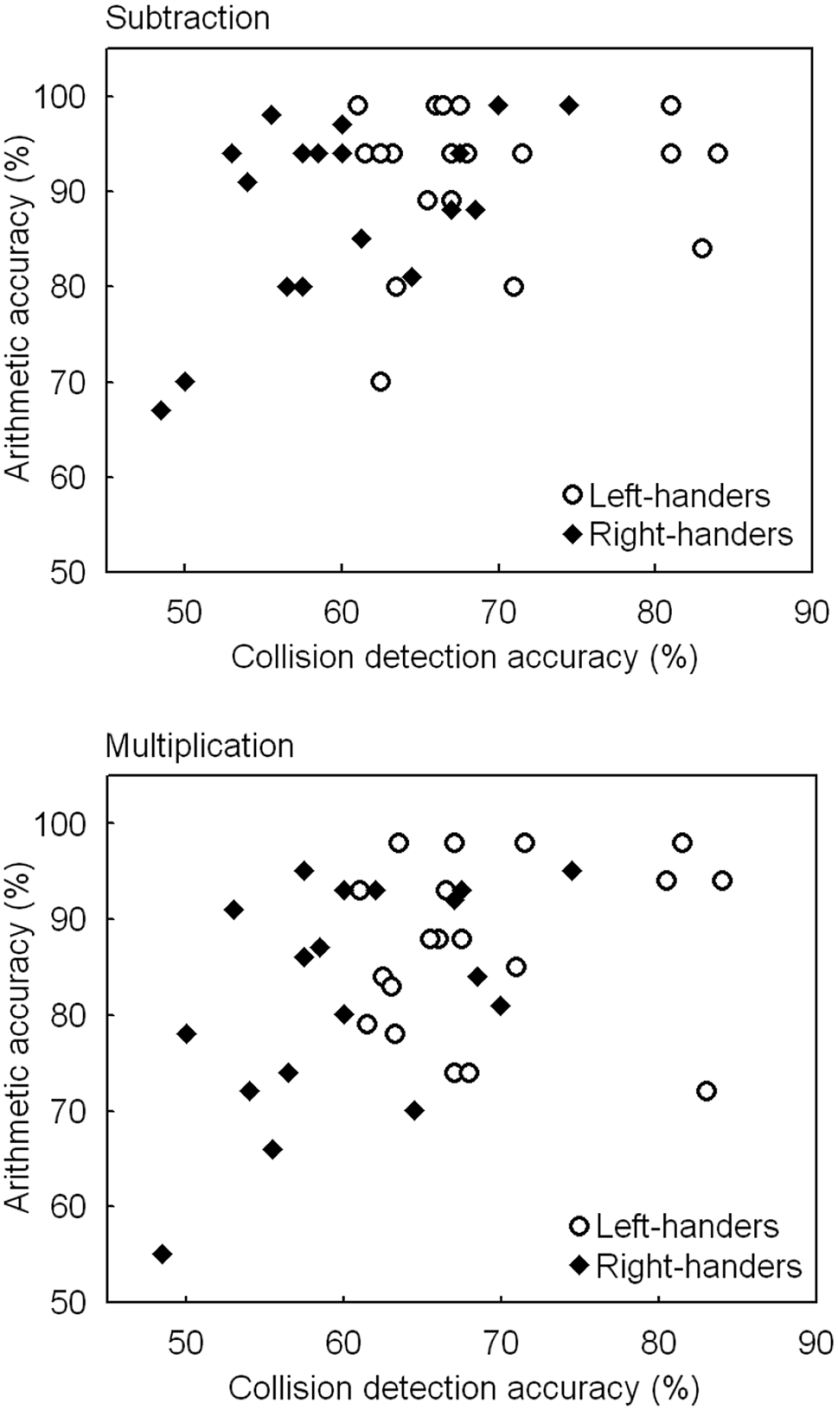
Scatter plot of the collision detection accuracy and arithmetic accuracy scores, illustrating a positive association. The accuracy scores represent the collision conditions (with and without distractor) and the arithmetic conditions of subtractions (top panel) and multiplications (lower panel)

Regression analysis was conducted to determine how handedness affected behaviour at the individual level across both handedness groups. In assessing both tasks, we observed that collision detection accuracy and arithmetic accuracy showed a positive association for subtractions (top panel, Fig. 6) and multiplications (lower panel, Fig. 6) as a function of handedness. The regression analyses for right-handers revealed significant outputs for subtractions, *F*(1, 35) = 6.2, *p* < 0.03, with *β* = 0.53 and *R*^*2*^ = 0.28, suggesting that 28% of the variance can be explained by the model (adjusted *R*^*2*^ = 0.24) and for multiplications, *F*(1, 35) = 5.42, *p* < 0.05, with *β* = 0.50 and *R*^*2*^ = 0.25, suggesting that 25% of the variance can be explained by the model (adjusted *R*^*2*^ = 0.21). For left-handers, no significant outputs were observed, *p* > 0.05. In addition, no effects were observed for the collision detection time and arithmetic accuracies, *p* > 0.05.

## Discussion

Making predictions is an innate capability of the human brain. In particular, the brain makes sense of the environment by predicting future events and by testing whether these are in line with incoming sensory information and previous experiences (Clark, 2013; Schubotz, 2007). To form these predictions, the dimensions of space, time and number are elementary. That is, a representation can be created by knowing where, when and how many, enabling us to respond to and learn about environmental regularities (Burr, et al. 2010; Lourenco & Longo, 2011; Winter, et al. 2015).

Predictive behaviour, such as estimating collisions, is crucial to our everyday activities, such as anticipating the course of moving objects and (de)synchronising our actions with them (Enns & Lleras, 2008; Senot, et al. 2003). We tested this real-world scenario by asking participants to detect whether collisions between moving objects would occur or not; decisions that are made on the basis of the use of spatial and temporal information from the motion trajectories, guided by attention to space and time. In addition, the participants completed arithmetic tests of addition, subtraction and multiplication, which are common in daily life such as required for counting and formal mathematics. In this study, we examine interdependencies between space–time–number by assessing both tasks in a group of left- and right-handers due to their distinct sensorimotor experiences.

### Handedness group profiles: space–time and number processing

Handedness is a manifestation of brain lateralisation that provides a representational index of the hands and that captures preference for manual activities (Corballis & Häberling, 2017). Our results from the collision detection task revealed that both handedness groups showed distinct behavioural performances, with left-handers being more accurate than right-handers; a performance advantage that could be due to a greater range of sensorimotor experiences and space–time integration pathways between both hemispheres (Assmus, et al. 2003; Cherbuin & Brinkman, 2006; Serrien, et al. 2012). In this context, hand use and sensorimotor competence are bi-directionally linked, shaping the information processing and associations between sensorimotor and attentional systems (Buckingham, et al. 2011; Le Bigot & Grosjean, 2012). Moreover, handedness affects the representation of extra-personal and peripersonal space with left-handers showing bilateral hemispheric activity whereas right-handers demonstrate an asymmetry of both hemispheres (Colman, et al. 2017; O’Regan & Serrien, 2018). Further differences between left- and right-handers have been proposed with distinct neglect-like patterns as a result of alertness-related modulations. In particular, Bareham et al. (2015) observed that left-handers experienced a leftward hemispheric shift in attention with drowsiness whereas right-handers have the opposite pattern, a distinction that could be due to differences in the attentional mechanisms that control alertness and direct attention to external stimuli (Liu, et al. 2009). In this study, we modified the attentional demands of the object collision task by means of distractors. We observed that their presence slowed the detection time across all participants, suggesting that difficult decisions take more time than easier ones and engage more neural circuitry for optimising behaviour (Assmus, et al. 2005; Smout, et al. 2019; Spapé & Serrien, 2011).

To take into account differences due to personality traits, we included the RST-PQ (Corr & Cooper, 2016); a questionnaire that associates personality with distinct brain systems labelled as BIS that predicts an individual's response to anxiety and performance avoidance as opposed to BAS that supports motivation and desired outcomes. Previous work has shown that left-handers have higher BIS scores than right-handers, which has been coupled with different levels of negative affect (Beaton, et al. 2017; Hardie & Wright, 2014). However, we observed no significant differences between left- and right-handers for any of the subtests, suggesting that there were no distinct variations in personality traits in our sample.

We noted no clear pattern of arithmetic performance differences between both handedness groups; a topic that has provided mixed claims throughout the literature (Annett & Kilshaw, 1982; Cheyne, et al. 2010; Crow, et al. 1998). While some studies have shown that left-handers are strong in mathematics and consistent right-handers perform least, others have suggested that mixed-handers are more disadvantaged. In a more recent study, Sala, et al. (2017) concluded that the relationship between handedness group profiles and mathematical ability is complex and depends on several factors, such as age, gender and type of task. This conclusion is in line with evidence that changes in mathematical processing occur as a function of development, and that arithmetic achievement depends on domain-general as well as domain-specific knowledge (Arsalidou & Taylor, 2011).

### Object collisions, arithmetic calculations and individual differences

Theories that account for functional overlap of space–time–number processing suggest the use of shared resources, common formats or a reliance on cross-dimensional mappings (Fias et al., 2003; Lakoff & Johnson, 1980; Walsh, 2003). In this respect, parietal circuits take a central role for the processing of space, time and number (Bjoertomt, et al. 2002; Coull & Nobre, 1998; Eger et al., 2009) as well as for the interactions between space–time (Magnani, et al. 2010; Oliveri, et al. 2009), space–number (Hubbard, et al. 2005; Oliveri, et al. 2004), and time-number (Burr, et al. 2010; Hayashi, et al. 2013). Of note is, however, that each hemisphere responds to specific task characteristics. In particular, for space and time processing, there is greater sensitivity of right inferior parietal circuitry for orienting in space (O’Reilly, et al. 2008) versus left inferior parietal circuitry for cueing in time and for space–time integration (Assmus, et al. 2003; Coull & Nobre, 2008). For number processing and numerical operations, inferior parietal regions are usually activated across both hemispheres, with specificity according to the type and complexity of the problem (Arsalidou & Taylor, 2011). However, there is shared circuitry across basic numerical and arithmetic tasks that is located within the left hemisphere, i.e., intraparietal sulcus alongside precentral areas. Therefore, a left fronto-parietal circuit can be considered a core network of numerical knowledge in adults (Pesenti, et al. 2000; Simon, et al. 2004; Zago, et al. 2001). The relevance of this network is that it overlaps with sensorimotor circuitry that is recruited for predictive control related to own actions and external perceptual events (Coull, et al. 2008; O’Reilly, et al. 2008; Schubotz, 2007). Moreover, this type of prediction arises when the essential information concerns dynamic forward change with coding of transitions in space–time, and underscores a key role of the sensorimotor system for the prediction of future states within the adopted reference frame, be it the body or the environment (Schubotz, 2007).

The regression analysis revealed a positive relationship between the detection accuracy of object collisions and the performance of arithmetic calculations, albeit as a function of handedness. That is, an association was observed only in right-handers, suggesting a connection between manual lateralisation and arithmetic. Support for such a relationship comes from finger counting, which represents a natural routine that supports the acquisition of basic numerical and arithmetic principles (Butterworth, 1999). In Western cultures, counting involves a preferred starting-hand alongside a relative order of finger counting within a single hand. Thus, finger counting strategies that are shaped by sensorimotor experience and developed during childhood may influence and steer how numbers are represented and processed later in life (Fischer, 2008; Pesenti, et al., 2000). In adults, these hand-starting preferences have been observed to be different for left- and right-handers (Zago & Badets, 2016). That is, consistent left-handers typically started counting with their left hand whereas the opposite pattern was noted for consistent right-handers; a manual preference that aligned with their dominant hand for unimanual activities. Furthermore, an fMRI study demonstrated that left-starters showed higher activation in the right right-sided motor and premotor cortices when they perceived small numbers whereas right-starters showed the reverse pattern (Tschentscher, et al. 2012). Thus, handedness modulates the structural arrangement of finger counting routines and further influences the involvement of the motor-dominant hemisphere for number processing (Artemenko, et al. 2020). This reliance on effector-specific circuitry in left- and right-handers has also been observed for skilled movements, such as grasping (Martin, et al. 2011). Together, the findings suggest that hemispheric lateralisation of key brain regions distinctively guides the covariation of functions that cross cognitive domains.

Besides handedness, we also noted that the type of arithmetic operation played a role in the relationship between collision detection and calculation performances. In particular, the results showed that stronger space–time computations resulted in increased performances for subtraction and multiplication calculations. No effect was observed for additions, which could be due to the fewer demands on number processing as compared to the other tasks which likely required additional processing steps (Fehr, et al. 2007). In using basic arithmetic operations (addition, subtraction, multiplication), we observed increasingly longer response times and lower accuracy rates, confirming changes in complexity requirements. In our study, the results did not show that the collision detection time significantly linked with arithmetic performance, which is in line with research that has shown that people alter where rather than when they would hit targets, if given the choice (Brenner, et al. 2015).

In conclusion, space, time and number are key dimensions that underlie how we perceive, identify and act within the environment. In this study, we examined interdependencies between these dimensions using an object collision task that required space–time processing and arithmetic tests that involved number processing in left- and right-handers. Handedness of the participants influenced collision detection with left-handers being more accurate than right-handers, which is in line with the premise that hand preference guides individual differences as a result of sensorimotor experiences and distinct interhemispheric integration patterns. The data further showed that successful collision detection was a predictor for arithmetic achievement, at least in right-handers. These findings suggest that handedness plays a mediating role in binding information processing across domains, likely due to selective connectivity properties within the sensorimotor system that are guided by hemispheric lateralisation patterns.

## Data Availability

The data have been stored in the Open Science Framework data repository (https://osf.io/rq8nu/).
